# Impact of unilateral salpingectomy on ovarian reserve and long-term reproductive outcomes in reproductive-aged women

**DOI:** 10.3389/fendo.2026.1952475

**Published:** 2026-10-12

**Authors:** Yanna Zhou, Yiting Yang

**Affiliations:** Department of Obstetrics and Gynecology, Jinshan Branch of Shanghai Sixth People’s Hospital, Shanghai, China

**Keywords:** ovarian reserve, recurrent ectopic pregnancy, reproductive potential, salpingectomy, tubal pregnancy

## Abstract

**Objective:**

To investigate the short-term impact of unilateral salpingectomy on ovarian reserve and to evaluate its comprehensive value regarding long-term reproductive outcomes in women of reproductive age.

**Methods:**

In this prospective cohort study, 96 reproductive-aged women diagnosed with ectopic pregnancy (EP) were enrolled. Base on the surgical and therapeutic regimens, participants were allocated into a study group (undergoing laparoscopic unilateral salpingectomy, *n* = 48) and a control group (preservation of the fallopian tube, including either laparoscopic salpingostomy or systemic methotrexate [MTX] therapy, *n* = 48). Biochemical markers of ovarian reserve (anti-Müllerian hormone [AMH], serum follicle-stimulating hormone [FSH], etc.) and morphological parameters (ovarian volume and antral follicle count [AFC]) were dynamically monitored and compared between the two groups at baseline and at 1, 3, and 6 months postoperatively. All participants were followed up for 24 months to compare spontaneous ovulation rates, spontaneous pregnancy rates, and the incidence of recurrent EP.

**Results:**

The study group exhibited transient fluctuations in biochemical markers indicative of diminished ovarian reserve in the early postoperative period (1 month), which normalized by 6 months postoperatively. Regarding morphological compensation, the contralateral ovarian volume and AFC in the study group increased significantly at 1–3 months postoperatively compared with the control group (*P*< 0.05); however, these parameters converged between the two groups by 6 months (*P*>0.05). Over the 24-month follow-up, there were no statistically significant differences in spontaneous ovulation or spontaneous pregnancy rates between the groups (*P*>0.05). Notably, the recurrence rate of EP in the study group was 2.08% (1/48), significantly lower than the 16.67% (8/48) observed in the control group (*P*< 0.05).

**Conclusions:**

Unilateral salpingectomy induces a transient early decline in ovarian reserve, counterbalanced by a distinct short-term compensatory response in the contralateral ovary. Crucially, this procedure does not compromise long-term natural conception potential while significantly mitigating the risk of recurrent EP. For patients with normal ovarian reserve and a healthy contralateral tube, unilateral salpingectomy represents a safe, effective, and optimal treatment strategy for maximizing protection against recurrent EP.

## 1 Introduction

Tubal ectopic pregnancy (EP) is characterized by the implantation and subsequent development of an embryo within the fallopian tube, which accounts for over 95% of all ectopic pregnancies. It represents a primary contributor to maternal morbidity and mortality associated with pregnancy (1). In recent years, EP rates have shown a continuous upward trend on a global scale (2). Coupled with the introduction of China’s pro-natal policies, an increasing number of women—particularly those of advanced maternal age, with a history of cesarean delivery, or with prior EP—who have a strong desire for future fertility now face dual challenges regarding reproductive health and safety in their family planning decisions (3). As the critical conduit for gamete encounter and early embryonic transport, the fallopian tube’s anatomical integrity and physiological function are directly linked to natural conception capacity (4). Conventional salpingectomy, as the standard procedure for definitively treating tubal EP and salvaging the patient’s life, has long been the focus of controversy in obstetrics and gynecology regarding its potential impact on ipsilateral ovarian reserve and long-term reproductive potential (5). Reproductive potential encompasses not only normal ovarian hormone secretion and ovulation but also the capacity to achieve a subsequent successful pregnancy and deliver a healthy neonate (6). Therefore, on the premise that the disease is effectively cured, how to maximize the protection of reproductive function in women of childbearing age has become one of the key metrics for evaluating the merits of therapeutic strategies for tubal EP (7).

To date, conclusions regarding the impact of salpingectomy on ovarian reserve and reproductive outcomes remain controversial. Some scholars argue that, while removing the lesion, salpingectomy may damage the arterial arcades within the mesosalpinx, thereby reducing ipsilateral ovarian blood supply, suppressing ovarian hormone synthesis and folliculogenesis, and potentially leading to diminished ovarian reserve and impaired natural conception rates in women of reproductive age (8–10). Thus, systematic assessment of postoperative ovarian reserve carries significant clinical value. The present study aims to systematically evaluate the effect of unilateral salpingectomy on the reproductive potential of women of childbearing age, addressing the limitations of existing studies in terms of sample size and follow-up duration, so as to provide more scientific and comprehensive evidence-based support for optimizing the management strategy for tubal EP.

## 2 Materials and methods

### 2.1 Study population

This prospective cohort study recruited women of reproductive age with unilateral tubal EP who wished to preserve fertility, all of whom were admitted to the Department of Gynecology at Shanghai Sixth People’s Hospital Jinshan Branch between January 2022 and December 2023. Diagnosis was established in every case via serial serum β-human chorionic gonadotropin (β-hCG) measurements and transvaginal ultrasonography, strictly following standard diagnostic protocols. The study protocol received approval from the Ethics Committee of Shanghai Sixth People’s Hospital Jinshan Branch (Approval No. jsxyy202117), and all participants provided written informed consent prior to their inclusion in the study.

Inclusion Criteria: (1) Aged 20–40 years with a clearly stated plan for conception in the near future; (2) Preoperative transvaginal ultrasound confirming unilateral tubal EP, with no apparent abnormalities in the contralateral fallopian tube or either ovary upon inspection; (3) No history of ovarian surgery, pelvic radiotherapy, or known premature ovarian insufficiency (POI); (4) Baseline serum anti-Müllerian hormone (AMH) ≥1.1 ng/mL and regular menstrual cycles (25–35 days).

Exclusion Criteria: (1) Presence of severe pelvic adhesions, endometriosis (stage III–IV), or ovarian tumors; (2) History of prior EP or unilateral/bilateral salpingectomy; (3) Pre-existing endocrine disorders (e.g., polycystic ovary syndrome, thyroid dysfunction, hyperprolactinemia); (4) Intraoperative findings revealing significant organic abnormalities in the contralateral fallopian tube or ovaries; (5) Loss to follow-up or incomplete medical records postoperatively.

### 2.2 Grouping and study protocol

Patients meeting the inclusion criteria were allocated into two groups based on the final treatment received, determined by shared decision-making between the patient and clinicians: the study group (underwent laparoscopic unilateral salpingectomy) and the control group (preservation of the fallopian tube, including either laparoscopic salpingostomy or systemic methotrexate [MTX] therapy). Baseline data were collected for all participants prior to treatment, including age, gravidity and parity, history of EP, preoperative serum β-hCG levels, maximum gestational sac diameter, and ovarian ultrasound parameters.

The participant enrollment flow is illustrated in **Figure 1**. Of the 132 patients with EP initially screened between January 2022 and December 2023, 58 were scheduled for salpingectomy and 74 for conservative treatment. Following rigorous application of the inclusion and exclusion criteria, 7 patients were excluded from the planned salpingectomy group (2 with severe pelvic adhesions, 3 with prior salpingectomy, and 2 with endocrine disorders), and 9 were excluded from the conservative treatment group (3 with severe pelvic adhesions, 4 with prior salpingectomy, and 2 with endocrine disorders). To further minimize confounding bias, 1:1 propensity score matching (PSM) was performed using age, body mass index (BMI), and gravidity/parity as covariates. This yielded a final analytical cohort of 96 patients, with 48 in the study group and 48 in the control group.

**Figure 1:**
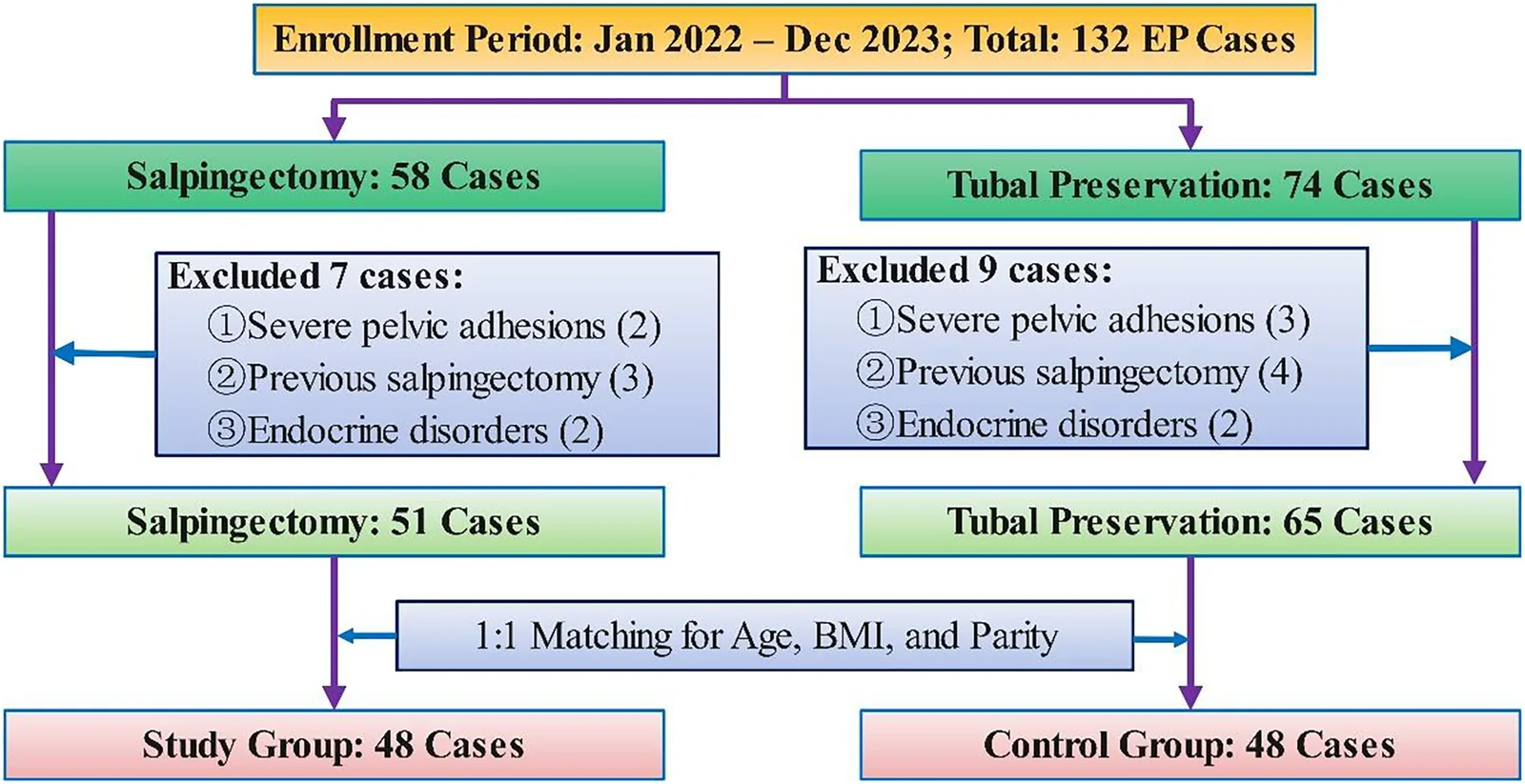
Workflow of patient enrollment and screening.

### 2.3 Outcome measures and assessment methods

(1) Evaluation of Ovarian Reserve: Peripheral venous blood samples were collected after an overnight fast at four time points: preoperatively (on the day of EP diagnosis) and postoperatively at 1, 3, and 6 months. Postoperative sampling was uniformly performed on days 2–4 of the menstrual cycle. Serum concentrations of follicle-stimulating hormone (FSH), luteinizing hormone (LH), estradiol (E_2_), and AMH were measured. Concurrently, a color Doppler ultrasound examination was performed by the same experienced sonographer. The longitudinal, transverse, and anteroposterior diameters of both ovaries were measured to calculate ovarian volume using the formula: *Volume = length × width × thickness × 0.523*. The total number of antral follicles measuring 2–9 mm in diameter (antral follicle count, AFC) was also recorded for each ovary.

(2) Follow-up of Reproductive Outcomes: Participants were followed up for a minimum of 24 months via a combination of outpatient clinic visits and telephone interviews. The primary endpoints included: (i) spontaneous ovulation rate (confirmed by basal body temperature charts and ovulation predictor kits); (ii) spontaneous pregnancy rate (defined as clinical pregnancy, evidenced by the presence of fetal cardiac activity on ultrasonography); and (iii) recurrence rate of EP. At discharge, all participants, irrespective of treatment allocation, were uniformly advised to practice effective contraception for three months after serum β-hCG had returned to non-pregnant levels, so as to allow complete physical and endocrine recovery before attempting conception. This advice was identical in the two groups and was not dependent on the treatment modality received (salpingectomy, salpingostomy, or systemic methotrexate). Consequently, any delay in conception attributable to this recommendation was non-differential between the groups and therefore should not be regarded as a confounding factor in the between-group comparison of reproductive outcomes. Additionally, the time interval (in months) from the date of serum β-hCG normalization to the first documented clinical pregnancy was recorded.

### 2.4 Statistical analysis

Statistical analyses were conducted using SPSS software (version 26.0; IBM Corp., Armonk, NY, USA). Continuous variables with normal distribution are presented as mean ± standard deviation (SD, $\bar{x}$ ± s). Between-group comparisons were performed using the independent samples *t*-test or the Mann–Whitney *U*test (11) for non-normally distributed data. Longitudinal changes in repeated measures were analyzed using repeated-measures analysis of variance (ANOVA), with *post-hoc* pairwise comparisons conducted using the least significant difference (LSD) method. Categorical variables are expressed as frequencies (percentages) and compared using the chi-square (χ^2^) test or Fisher’s exact test (12) where appropriate. Kaplan–Meier survival curves were constructed to estimate cumulative pregnancy rates, and intergroup differences were assessed using the log-rank test. A two-tailed *P*-value< 0.05 was considered statistically significant for all analyses.

## 3 Results

### 3.1 Baseline homogeneity of demographic and clinicopathological characteristics

We conducted a systematic assessment of baseline homogeneity among all enrolled women of reproductive age with unilateral tubal EP. Intergroup comparisons between the study groupand the control group revealed a high degree of balance across both demographic characteristics and key clinicopathological parameters.

Analyses using the independent samples *t*-test and chi-square (*χ²*) test showed no statistically significant intergroup differences in mean age at initial presentation or BMI. With respect to obstetric history, gravidity and parity were highly comparable between the two groups, ruling out selection bias arising from prior pregnancy profiles that could confound subsequent evaluations of ovarian function.

Furthermore, no significant intergroup differences were observed in duration of amenorrhea, maximum diameter of the pelvic mass, or baseline peripheral serum β-hCG levels (all *P*> 0.05). The alignment of baseline disease characteristics between the two cohorts eliminated selection bias associated with major clinical variables, providing a robust and reliable foundation for parallel control in subsequent longitudinal follow-up analyses. Detailed baseline characteristics are summarized in **Table 1**.

**Table 1:** Comparison of baseline characteristics between the two groups.

Item	Study group (n=48)	Control group (n=48)	t/χ²	P
Age (years)	26.82 ± 6.90	27.92 ± 7.61	0.742	0.460
Gravidity	2.52 ± 0.84	2.32 ± 0.71	1.260	0.211
Parity	1.34 ± 0.59	1.22 ± 0.49	1.084	0.281
BMI (kg/m²)	22.82 ± 5.83	23.87 ± 3.22	1.092	0.278
Duration of amenorrhea (days)	52.57 ± 3.23	51.21 ± 4.52	1.696	0.093
Maximum diameter of pelvic mass (mm)	28.23 ± 16.13	26.78 ± 18.70	0.407	0.685
Baseline serum β-hCG (mIU/mL)	3829.35 ± 2963.45	3325.15 ± 2845.41	0.850	0.397

### 3.2 Longitudinal dynamic trajectories of early postoperative ovarian endocrine profiles and specific reserve biomarkers

#### 3.2.1 Longitudinal and cross-sectional interaction analyses of gonadotropin (FSH and LH) levels

Serial follow-up assessments of peripheral serum FSH and LH levels were conducted at 1, 3, and 6 months after normalization of post-treatment serum β-hCG. Repeated-measures ANOVA revealed significant intergroup differences in the temporal trajectories of serum FSH and LH levels (presented as $\bar{x}$ ± s, unit: U/L; all *P*< 0.05). Detailed data are provided in **Table 2**.

**Table 2:** Comparative analysis of serum FSH and LH levels between the two groups at 1, 3 and 6 months post-treatment (U/L, $\bar{x}$ ± s).

Group	FSH (U/L)			LH (U/L)		
	1 Month	3 Months	6 Months	1 Month	3 Months	6 Months
Control Group (n=48)	6.69 ± 2.71	6.81 ± 2.80	6.41 ± 2.41	6.10 ± 2.59	5.79 ± 2.10	6.50 ± 2.71
Study Group (n=48)	10.00 ± 4.82	9.11 ± 3.77	8.30 ± 3.49	8.74 ± 3.56	9.42 ± 3.09	7.96 ± 2.53
t	4.417	3.393	3.087	4.155	6.732	2.728
P	<0.001	0.001	0.003	<0.001	<0.001	0.008

Within-group paired longitudinal analyses (with postoperative measurements referenced to each patient’s individual preoperative baseline) revealed markedly divergent evolutionary trajectories between the two cohorts. Throughout the entire follow-up window (postoperative 1, 3, and 6 months), the control group exhibited no statistically significant fluctuations in serum FSH or LH concentrations relative to baseline (all P> 0.05). In contrast, the study group showed significantly elevated FSH and LH levels at 1 and 3 months postoperatively compared with preoperative values (both P< 0.05); by the 6-month postoperative timepoint, these levels were statistically indistinguishable from preoperative baselines (P> 0.05). Detailed comparative data are provided in **Table 3**.

**Table 3:** Intragroup longitudinal analysis of serum FSH and LH levels from pre-operative baseline to different post-operative time points in the two groups ( $\bar{x}$ ± s).

Group	Time point	FSH (U/L)			LH (U/L)		
Control Group (n=48)	Post-op	1 Month	3 Months	6 Months	1 Month	3 Months	6 Months
		6.69 ± 2.71	6.81 ± 2.80	6.41 ± 2.41	6.10 ± 2.59	5.79 ± 2.10	6.50 ± 2.71
	Pre-op	6.71 ± 2.05			6.08 ± 2.36		
	T	-0.041	0.200	-0.657	0.040	-0.636	0.810
	P	0.968	0.842	0.513	0.969	0.526	0.420
Study Group (n=48)	Post-op	1 Month	3 Months	6 Months	1 Month	3 Months	6 Months
		10.00 ± 4.82	9.11 ± 3.77	8.30 ± 3.49	8.74 ± 3.56	9.42 ± 3.09	7.96 ± 2.53
	Pre-op	7.22 ± 1.94			7.24 ± 1.62		
	T	3.707	3.088	1.874	2.657	4.329	1.660
	P	<0.001	0.003	0.064	0.009	<0.001	0.100

#### 3.2.2 Damage and recovery kinetics of ovarian-specific reserve biomarkers (AMH and E_2_)

Cross-sectional intergroup comparisons demonstrated that at 1 and 3 months postoperatively, serum E_2_ and AMH levels in the study group were significantly lower than those in the control group (both *P*< 0.05), with this intergroup difference no longer reaching statistical significance at 6 months postoperatively (*P*> 0.05). Detailed data are provided in **Table 4**.

**Table 4:** Comparison of serum E_2_ and AMH levels between the two groups of tubal EP patients at 1, 3, and 6 months after serum β-hCG normalization ( $\bar{x}$ ± s).

Group	E_2_ (pmol/L)			AMH (ng/mL)		
	1 Month	3 Months	6 Months	1 Month	3 Months	6 Months
Control Group (n=48)	128.22 ± 24.01	126.30 ± 23.78	129.28 ± 24.54	3.64 ± 0.87	3.83 ± 1.12	4.17 ± 1.76
Study Group (n=48)	111.44 ± 22.24	108.74 ± 21.67	120.85 ± 20.12	3.22 ± 0.85	3.37 ± 0.94	3.74 ± 1.24
t	3.552	3.781	1.840	2.392	2.180	1.384
P	0.001	<0.001	0.069	0.030	0.032	0.170

Within-group longitudinal trajectory analyses revealed that across all postoperative timepoints (1, 3, and 6 months), serum AMH and E_2_ levels in the control group showed no statistically significant deviations from their respective preoperative baselines (all P> 0.05). In contrast, the study group exhibited significant reductions in both AMH and E_2_ levels at 1 and 3 months postoperatively compared with preoperative values (all P< 0.05); by 6 months postoperatively, these biomarkers were no longer statistically distinguishable from preoperative baselines (P> 0.05). Detailed comparative data are provided in **Table 5**. Comparative profiles of serum FSH, LH, E_2_, and AMH levels across preoperative and postoperative timepoints in the two cohorts are illustrated in **Figure 2**.

**Table 5:** Intragroup longitudinal follow-up analysis of serum E_2_ and AMH levels from pre-operative baseline to different post-operative time points in the two groups ( $\bar{x}$ ± s).

Group	Time Point	E_2_ (pmol/L)			AMH (ng/mL)		
Control Group (n=48)	Post-op	1 Month	3 Months	6 Months	1 Month	3 Months	6 Months
		128.22 ± 24.01	126.30 ± 23.78	129.28 ± 24.54	3.64 ± 0.87	3.83 ± 1.12	4.17 ± 1.76
	Pre-op	128.56 ± 28.48				3.86 ± 1.46	
	T	-0.063	-0.422	0.133	-0.897	-0.113	0.930
	P	0.950	0.674	0.895	0.372	0.910	0.350
Study Group (n=48)	Post-op	1 Month	3 Months	6 Months	1 Month	3 Months	6 Months
		111.44 ± 22.24	108.74 ± 21.67	120.85 ± 20.12	3.22 ± 0.85	3.37 ± 0.94	3.74 ± 1.24
	Pre-op	126.50 ± 15.29			3.79 ± 1.04		
	T	-3.867	-4.640	-1.549	-2.940	-2.076	-0.214
	P	<0.001	<0.001	0.125	0.004	0.041	0.831

**Figure 2:**
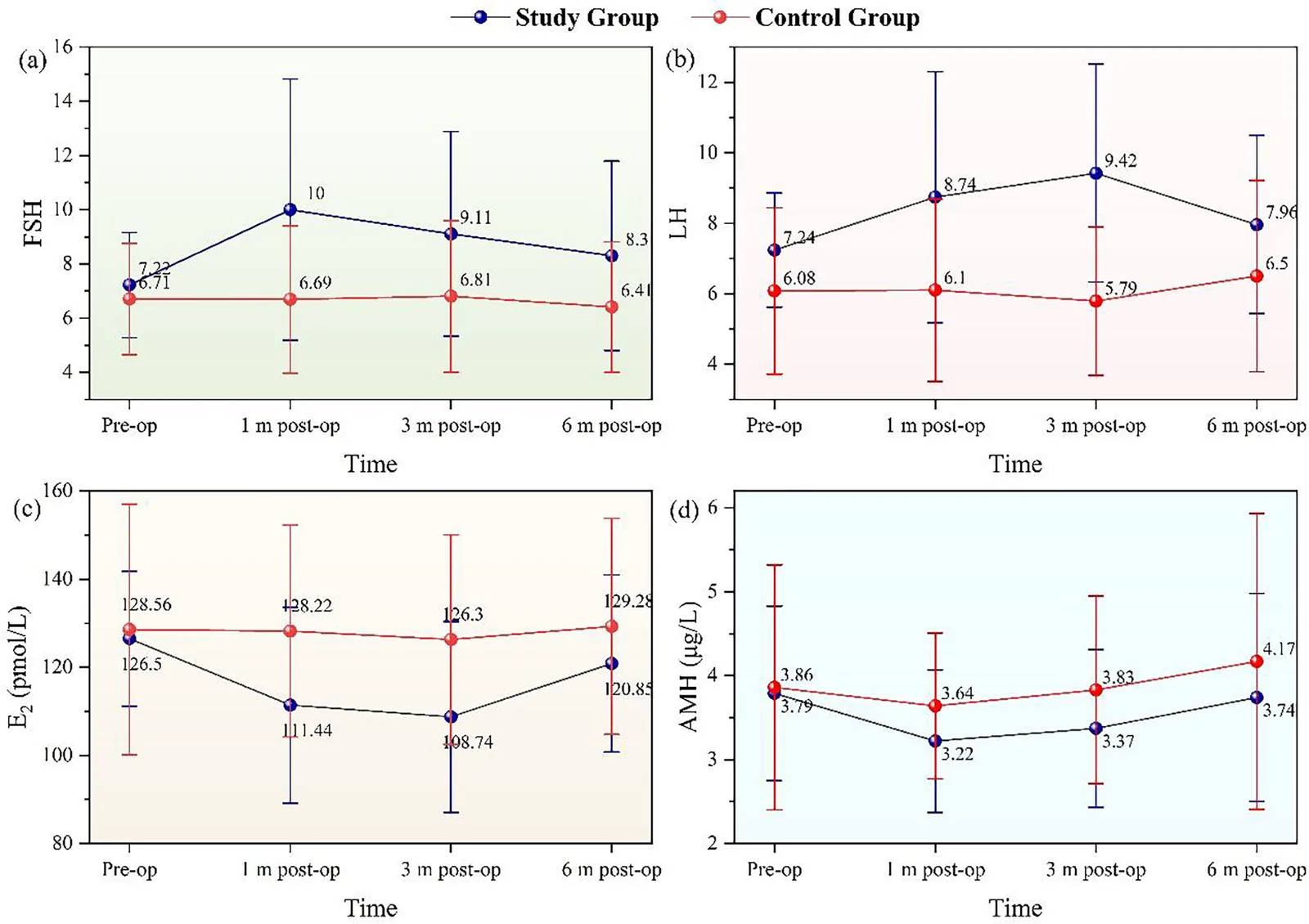
Dynamic changes in serum gonadotropins and ovarian reserve biomarkers following unilateral. **(a)** FSH. **(b)** LH. **(c)** E_2_. **(d)** AMH.

### 3.3 Ultrasonographic evidence of structural and morphological compensatory mechanisms in the contralateral ovary

Longitudinal within-group analyses revealed that in the study group, contralateral ovarian volume was significantly enlarged at 1 and 3 months postoperatively compared with the preoperative baseline (both *P*< 0.05), accompanied by a statistically significant increase in AFC over the same timepoints (both *P*< 0.05). By 6 months postoperatively, no statistically significant intergroup differences were detected in contralateral ovarian volume or AFC between the study group and the control group (both *P*> 0.05). Notably, the control group exhibited no statistically significant variations in bilateral ovarian volume or AFC across all postoperative timepoints (1, 3, and 6 months; all *P*> 0.05). Detailed comparative data are provided in **Table 6**. Serial ultrasonographic monitoring findings of contralateral (study group) and bilateral (control group) ovarian volume and AFC across preoperative and postoperative timepoints in the two cohorts are illustrated in **Figure 3**.

**Table 6:** Ultrasonic dynamic comparison of contralateral/bilateral ovarian volume and AFC between the two groups at pre-operative and different post-operative time points ( $\bar{x}$ ± s).

Observation Index	Group	Pre-op	1 Month (Post-op)	3 Months (Post-op)	6 Months (Post-op)
Ovarian Volume (cm³)	Control Group (n=48)	6.72 ± 2.24	6.73 ± 2.37	6.81 ± 2.13	6.75 ± 2.44
	Study Group (Contralateral) (n=48)	6.87 ± 2.16	7.78 ± 2.34	7.94 ± 2.58	6.98 ± 2.25
	T	0.334	-2.184	2.340	0.480
	P	0.739	0.031	0.021	0.632
AFC (n)	Control Group (48)	7.13 ± 2.72	7.17 ± 2.75	7.21 ± 2.88	7.10 ± 2.81
	Study Group (Contralateral) (48)	7.24 ± 2.79	8.36 ± 2.98	8.48 ± 3.07	7.32 ± 2.91
	T	0.196	-2.043	2.090	0.377
	P value	0.845	0.045	0.039	0.707

**Figure 3:**
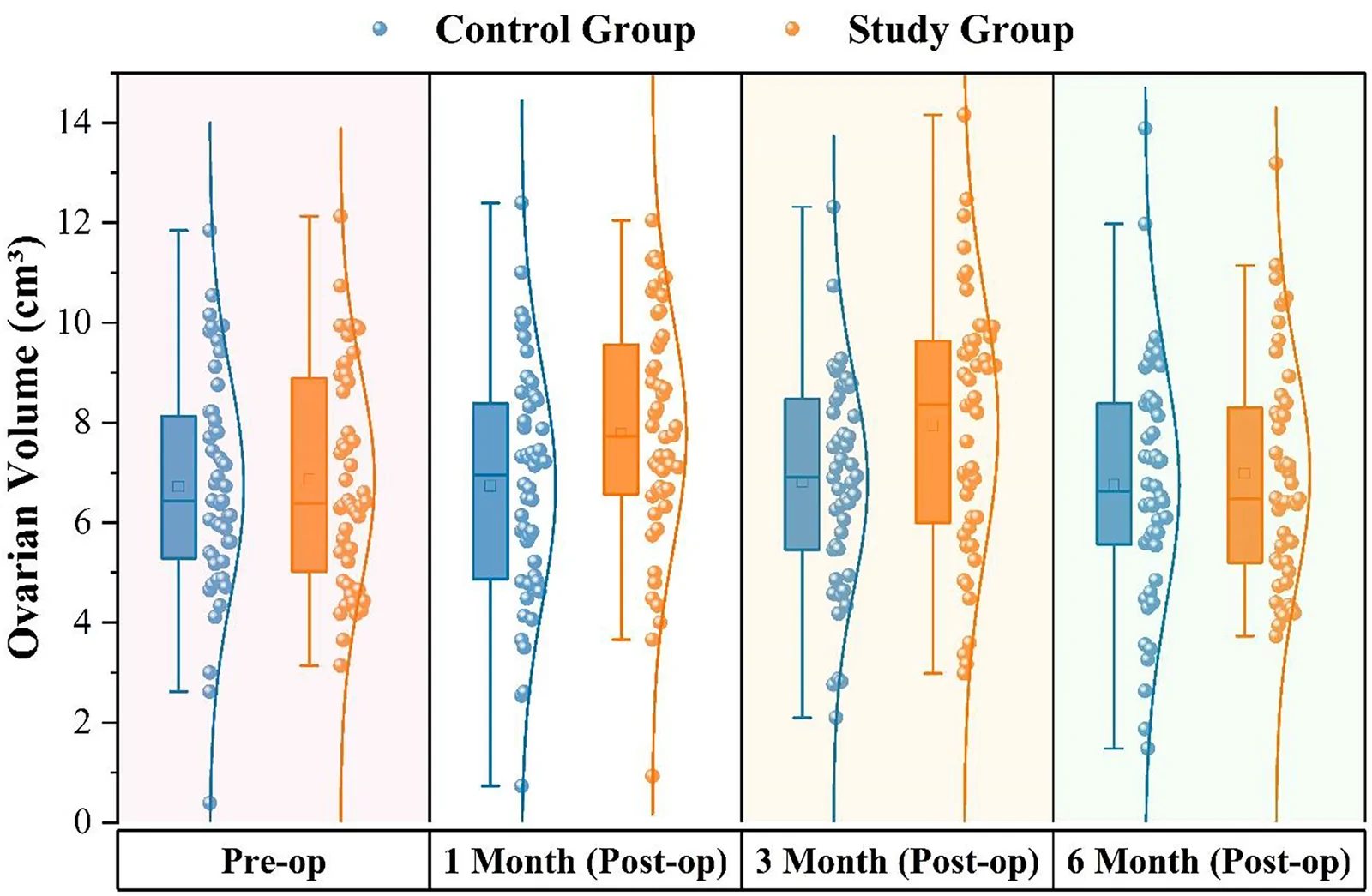
Morphological compensatory response of the contralateral ovary following unilateral salpingectomy.

### 3.4 Survival analysis of long-term spontaneous reproductive performance and recurrent EP risk

All enrolled participants completed the full 24-month post-treatment follow-up protocol.

#### 3.4.1 Spontaneous ovulation rate

The spontaneous ovulation rate within the 24-month post-treatment period was 89.58% (43/48) in the study group and 93.75% (45/48) in the control group, with no statistically significant intergroup difference (*P*> 0.05).

#### 3.4.2 Spontaneous pregnancy rate

During the entire follow-up period, the spontaneous pregnancy rate was 57.18% (26/48) in the study group and 60.41% (29/48) in the control group, with no statistically significant intergroup difference (*P*> 0.05). Kaplan–Meier survival analysis was performed to estimate cumulative pregnancy probability over time; the median time to first clinical pregnancy was 8.2 months (95% CI: 7.1–9.3) in the study group and 7.5 months (95% CI: 6.2–8.8) in the control group. The log-rank test revealed no statistically significant intergroup difference in cumulative pregnancy probability (*P*> 0.05).

#### 3.4.3 Recurrent EP rate

The recurrent EP rate was 2.08% (1/48) in the study group and 16.67% (8/48) in the control group, representing a statistically significant intergroup difference (*P*< 0.05). Among the recurrent cases in the study group, 1 occurred in the contralateral fallopian tube. Of the 8 recurrent cases in the control group, 7 occurred in the preserved ipsilateral fallopian tube and 1 in the contralateral tube, with 87.50% (7/8) of recurrent events arising from the originally affected and preserved tube (*P*< 0.05). Collectively, these data suggest that unilateral salpingectomy confers a significant protective effect against recurrent EP.

Comparative rates of spontaneous ovulation at 6 months post-treatment, cumulative pregnancy within 2 years post-treatment, and recurrent ectopic pregnancy between the two cohorts of patients with tubal ectopic pregnancy are illustrated in **Figure 4**.

**Figure 4:**
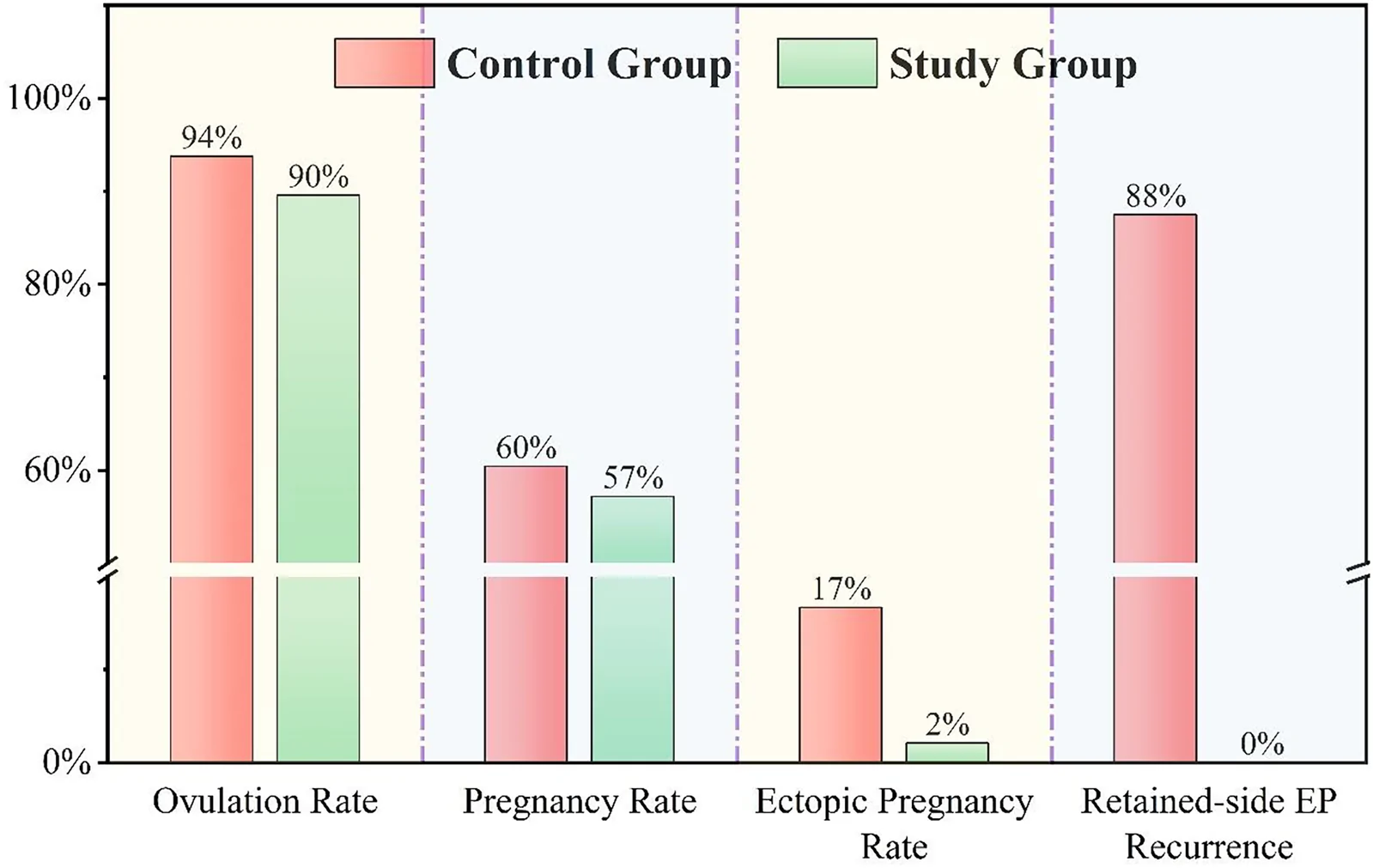
Long-term reproductive outcomes and cumulative recurrence rates of ectopic pregnancy over 24 months of follow-up.

## 4 Discussion

EP is one of the most prevalent gynecological emergencies among women of reproductive age, with tubal implantation accounting for the vast majority of cases (13–15). With the widespread adoption of minimally invasive surgical principles and growing emphasis on fertility preservation in reproductive medicine, the comparative efficacy of *salpingectomy* (resection of the affected tube) versus *fallopian tube conservative treatments* in terms of postoperative ovarian reserve and long-term reproductive outcomes remains a contentious topic in international reproductive endocrinology (16–18). Through a 24-month prospective longitudinal follow-up integrating microscopic endocrine biomarkers and macroscopic ultrasonographic morphometry, the present study comprehensively delineates the dynamic trajectory of ovarian function following unilateral salpingectomy—characterized by an initial transient impairment, followed by compensatory adaptation of the contralateral ovary, and culminating in complete functional remodeling. Our findings not only corroborate the safety of salpingectomy regarding long-term fertility preservation but also provide robust clinical evidence supporting the concept of an axial compensatory network among reproductive organs.

### 4.1 Short-term impact of salpingectomy on ovarian reserve and underlying mechanisms

The transient decline in ovarian reserve observed post-salpingectomy is conventionally attributed to iatrogenic injury to the arterial arcades within the mesosalpinx (19). The mesosalpinx harbors anastomotic branches between the ovarian and uterine arteries, which collectively supply a substantial portion of the ipsilateral ovarian blood flow. Transection of the mesosalpinx during surgery may compromise this vascular supply, thereby impeding folliculogenesis and steroidogenesis (20). The primary sources of ovarian perfusion include the ovarian branch of the uterine artery and the vascular network formed by anastomoses between the ovarian artery and branches within the mesosalpinx (21). Preserving the mesosalpinx while excising only the affected tubal segment theoretically mitigates disruption to the shared vasculature between the ovary and fallopian tube, thereby safeguarding ovarian reserve and potentially enhancing future assisted conception success rates (22, 23).

In the current study, cross-sectional analyses revealed that serum FSH and LH levels in the study group were significantly elevated compared with the control group at 1, 3, and 6 months following post-treatment β-hCG normalization (all *P*< 0.05). Similarly, serum AMH and E_2_ levels were markedly lower in the study group at 1 and 3 months postoperatively (both *P*< 0.05), with these intergroup differences resolving by 6 months (*P*> 0.05). Crucially, within-group longitudinal paired comparisons demonstrated that FSH, LH, AMH, and E_2_ levels in the study group were significantly higher at 1 and 3 months postoperatively relative to individual preoperative baselines (all *P*< 0.05), subsequently returning to preoperative levels by 6 months (*P*> 0.05). Conversely, the control group exhibited no statistically significant fluctuations in any endocrine parameter throughout the follow-up period relative to baseline (all *P*> 0.05). These data indicate that unilateral salpingectomy induces a transient early postoperative decline in ovarian reserve biomarkers (manifested biochemically as elevated FSH and reduced AMH/E_2_). This phenomenon likely reflects acute ischemia of the ipsilateral ovary following vascular compromise, leading to abrupt reductions in estrogen and inhibin secretion and consequent withdrawal of negative feedback on the hypothalamic-pituitary axis. Although gonadotropin levels in the study group exhibited a modest downward trend by 3 months postoperatively—coinciding with resolution of acute surgical stress—they remained elevated due to incomplete restoration of local ovarian microcirculation. Serial monthly monitoring unequivocally established that this impact is transient rather than indicative of permanent ovarian damage, aligning with conclusions from most contemporary large-scale investigations. For instance, Aytekin et al. (24) reported a significant reduction in AMH at 1 month following opportunistic salpingectomy (*p* = 0.043), which was not sustained at 3 months, concluding that salpingectomy exerts no deleterious effects on ovarian reserve, stromal blood flow, or sexual function. Similarly, Grynnerup et al. (25) found no significant short-term alterations in serum AMH levels following salpingectomy for EP, nor correlations between AMH dynamics and patient age or follow-up duration. In a cohort studied by Gay et al. (26), no significant inter-ovarian differences in ultrasonographic ovarian response to controlled ovarian hyperstimulation were observed between the surgically treated and contralateral ovaries in women undergoing *in vitro* fertilization (IVF) after unilateral salpingectomy for EP. While Koltyar et al. (27) acknowledged that most indications for salpingectomy do not adversely affect ovarian reserve, they noted conflicting data suggesting minor impairments in select studies primarily involving EP patients; nevertheless, they concluded that salpingectomy does not substantially diminish conception rates in women attempting natural pregnancy. Corroborating these findings, Morelli et al. (28), in a 2013 retrospective study involving 158 patients, demonstrated no significant intergroup differences in AMH, FSH, antral follicle count, or mean ovarian diameter between salpingectomy and non-salpingectomy cohorts. Our results reinforce these collective conclusions, affirming that the short-term impact of salpingectomy on ovarian reserve is clinically acceptable. Addressing concerns regarding intraoperative disruption of tissue architecture and vasculature, Venturella et al. (29) specifically investigated whether extensive resection of para-ovarian and para-tubal soft tissues during laparoscopic bilateral salpingectomy compromises ovarian reserve. Their findings indicated that even when the mesosalpinx is widely excised, salpingectomy per se does not impair ovarian reserve parameters..

### 4.2 Compensatory response of the contralateral ovary

Unilateral salpingectomy disrupts the anatomical continuity of the ipsilateral adnexa and alters the local microenvironment of the ipsilateral ovary, making early parallel morphological tracking of the affected side inherently challenging. To comprehensively and objectively characterize endogenous structural self-regulatory responses following unilateral gonadal insult, we conducted rigorous cross-sectional comparative analyses of morphological parameters, using high-resolution transvaginal Doppler ultrasound to evaluate the contralateral ovary in the study group and average bilateral ovarian metrics in the control group.

Our experimental data unequivocally demonstrate a robust morphological “compensatory hyperfunction” of the contralateral ovary following parenchymal injury or microcirculatory suppression of the ipsilateral ovary. At preoperative baseline, intergroup comparisons revealed no significant differences in ovarian volume or AFC—a surrogate marker of baseline ovarian reserve—between the two cohorts (all *P*> 0.05). Notably, contralateral ovarian volume and AFC in the study group were significantly elevated relative to preoperative values at 1–3 months postoperatively, whereas no analogous changes were observed in the bilateral ovaries of the control group. This compensatory phenomenon has been preliminarily described in prior literature: Aytekin et al. (24) reported that opportunistic salpingectomy performed concurrent with hysterectomy exerted no deleterious effects on ovarian reserve, interstitial ovarian blood flow, or sexual function. In a study evaluating the impact of adnexal surgical history on IVF outcomes, Ho et al. (30) found no significant differences in IVF success rates between patients undergoing unilateral salpingectomy versus fertility-sparing tubal surgery, providing indirect evidence for post-surgical ovarian functional compensation. Our morphological data further quantify this compensatory response: contralateral ovarian volume increased by ~16% and AFC by ~18% relative to preoperative baselines. From an evolutionary perspective, the bilateral reserve capacity of ovarian function serves as a safeguard for reproductive recovery following unilateral injury. Importantly, this compensation extends beyond structural changes to encompass endocrine modulation: the contralateral ovary may enhance follicular recruitment efficiency by increasing FSH sensitivity and upregulating the expression of key growth factors such as insulin-like growth factor-1 (IGF-1) and AMH. Nevertheless, the upper limits and durability of this compensatory mechanism require further investigation. By 6 months postoperatively, contralateral ovarian parameters in the study group were comparable to those of the control group, indicating that compensatory hypertrophy gradually regresses to a homeostatic steady state.

### 4.3 Long-term reproductive outcomes: comparable pregnancy rates and significantly reduced recurrent EP risk

The most clinically impactful finding of our study is that, despite transient early postoperative alterations in ovarian reserve biomarkers, long-term reproductive outcomes (spontaneous ovulation and pregnancy rates) over the 24-month follow-up period did not differ significantly between the two groups. Rosen et al. (31) demonstrated that, among patients with tubal EP, medical management with MTX was associated with a higher live birth rate compared with surgical intervention; however, this benefit came at the cost of elevated risks of recurrent EP and increased healthcare utilization. Another randomized controlled trial comparing the cost-effectiveness of salpingostomy versus salpingectomy for tubal EP found no significant intergroup difference in spontaneous pregnancy rates within 36 months post-treatment (32). Yet salpingostomy incurred higher additional treatment costs due to risks of persistent trophoblast and recurrent EP. Collectively, these data suggest that unilateral salpingectomy does not materially compromise the likelihood of successful conception in women of reproductive age with normal baseline ovarian reserve.

In stark contrast, the recurrent EP rate was 2.08% (1/48) in the study group versus 16.67% (8/48, 7 of which occurred in the preserved ipsilateral fallopian tube) in the control group, a statistically significant intergroup difference (*P*< 0.05). This result aligns closely with the meta-analysis by Fernandez et al. (33), which reported a recurrent EP rate of 12.7% in the conservative surgery group versus 3.5% in the salpingectomy group (odds ratio [OR] = 0.28). Prior consensus holds that while conservative surgery preserves tubal anatomy, it carries risks of tubal scarring, adhesion formation, and impaired peristalsis, which predispose to recurrent EP. Existing evidence indicates that even after conservative procedures such as salpingostomy, recurrent EP rates range from 6%–26%, with persistent tubal pregnancy occurring in approximately 18% of cases (34–36). Song et al. (37) further confirmed that although fertility-sparing tubal surgery is generally safe and feasible, surgical modality significantly impacts long-term tubal patency. Xiang et al. (38) reported that laparoscopic salpingostomy failed to improve tubal patency in patients with postoperative tubal occlusion, a finding corroborated by Cheng et al. (39), who documented a tubal patency rate of only 36.6% following salpingostomy. Our data directly validate that recurrent EP in the conservative treatment group predominantly occurred in the preserved ipsilateral tube, indirectly confirming functional impairment of the affected tube after conservative surgery. Thus, for patients who have completed childbearing or have a healthy contralateral fallopian tube, salpingectomy offers a marked advantage in preventing recurrent EP (40).

### 4.4 Clinical implications and individualized decision-making

Synthesizing our findings, clinical decision-making for women of reproductive age with unilateral tubal EP who desire future fertility requires careful weighing of the trade-offs between salpingectomy and fallopian tube conservative management, with considerations integrating both ovarian reserve biomarkers and long-term real-world reproductive outcomes rather than ovarian reserve markers alone. We propose the following evidence-based recommendations:

1. For patients with a grossly normal contralateral fallopian tube and adequate bilateral ovarian reserve (e.g., AMH ≥ 1.5 ng/mL), salpingectomy represents a prudent and reliable option. This approach effectively reduces the risk of recurrent EP without negatively impacting long-term spontaneous pregnancy rates demonstrated by our 24-month follow-up data, despite the transient short-term fluctuations observed in ovarian reserve biomarkers (41).
2. For patients with a solitary remaining ovary, a history of prior contralateral salpingectomy, or diminished ovarian reserve (e.g., AMH< 1.0 ng/mL), fallopian tube conservative surgery (e.g., salpingostomy) may better preserve ovarian blood supply and residual conception potential. However, clinicians must provide thorough counseling regarding the significantly elevated risk of recurrent EP associated with this approach (42).
3. Intraoperative technique should prioritize meticulous dissection as close to the serosal layer of the fallopian tube as possible. Clamp placement should be restricted to the superior margin of the mesosalpinx to minimize thermal spread to the ovarian vasculature, thereby avoiding inadvertent injury to mesosalpingeal vessels and the suspensory ligament of the ovary (43). However, it should be noted that during emergency laparotomy, inadvertent entrapment of the ovarian vessels within the clamp may occur due to limited visualization or the urgent need for hemostasis, which could compromise the function of the ipsilateral ovary. Therefore, regardless of the surgical approach, strict adherence to careful identification of ovarian vessels and atraumatic clamp placement is essential to preserve ovarian reserve.

In summary, unilateral salpingectomy induces a transient early postoperative decline in ovarian reserve (manifested by elevated follicle-stimulating hormone FSH and reduced AMH and estradiol E_2_ levels) in women of reproductive age, coupled with compensatory hyperfunction of the contralateral ovary (44). Long-term spontaneous pregnancy rates are comparable between salpingectomy and conservative management, while the risk of recurrent EP is significantly attenuated following salpingectomy (45). Thus, for patients with normal ovarian reserve and a healthy contralateral fallopian tube, salpingectomy is a safe, effective first-line treatment that maximizes recurrence risk reduction without materially compromising reproductive potential (46). All clinical decisions must be individualized based on patient age, ovarian reserve status, and contralateral tubal anatomy.

## 5 Conclusions

This prospective cohort study systematically evaluated the impact of unilateral salpingectomy on reproductive potential in women of childbearing age, yielding the following core conclusions:

First, unilateral salpingectomy results in a transient, early postoperative (1–3 months) reduction in ovarian reserve, characterized by elevated serum FSH and reduced AMH and E_2_ levels. These parameters return to preoperative baselines by 6 months postoperatively, accompanied by compensatory increases in contralateral ovarian volume and AFC—confirming that the impact of this procedure on overall ovarian function is reversible and limited in scope.

Second, regarding long-term reproductive outcomes, there were no statistically significant intergroup differences in spontaneous ovulation or pregnancy rates between the salpingectomy and conservative management cohorts. Crucially, salpingectomy reduced the risk of recurrent EP from 16.67% (8/48) to 2.08% (1/48).

These findings support salpingectomy as a safe, effective treatment option for patients with normal ovarian reserve and a healthy contralateral fallopian tube, balancing minimal impairment of reproductive potential with maximal protection against recurrent EP. However, individualized risk-benefit assessment remains essential: for patients with diminished ovarian reserve or a solitary remaining ovary, the potential adverse effects of salpingectomy on ovarian blood supply warrant cautious consideration, and fallopian tube conservative surgery should be prioritized.

This study has several limitations. First, the non-randomized allocation design, while adjusted for baseline confounders via propensity score matching, may still introduce selection bias. Second, ovarian function assessment was limited to biochemical markers (FSH, LH, E_2_, AMH) and ultrasonographic parameters (ovarian volume, AFC); more precise metrics such as AMH receptor assays or ovarian biopsy were not incorporated. Third, while the 24-month follow-up captured the majority of spontaneous conception events, some patients may require longer intervals to achieve pregnancy. Fourth, we did not conduct subgroup analyses stratified by surgical technique (e.g., with vs. without mesosalpinx preservation). Fifth, although alterations in the tubal mucosal immune microenvironment have been implicated in the pathogenesis of tubal EP and its recurrence, this study assayed no immune parameter, and the relationship between the tubal mucosal immune microenvironment and recurrent EP remains to be clarified.

Future research should validate our findings using larger-scale, multicenter randomized controlled trials with extended follow-up durations, in which mucosal immune assessment (e.g., cytokine and immune cell characterization of tubal lavage or mucosal samples) is incorporated, so as to clarify how local immune dysfunction of the affected tube relates to the risk of recurrent EP.

## Data Availability

The raw data supporting the conclusions of this article will be made available by the authors, without undue reservation.
